# The data availability landscape in seven sub-Saharan African countries and its role in strengthening sugar-sweetened beverage taxation

**DOI:** 10.1080/16549716.2020.1871189

**Published:** 2021-04-20

**Authors:** Agnes Erzse, Safura Abdool Karim, Anne Marie Thow, Gemma Ahaibwe, Hans Justus Amukugo, Gershim Asiki, Lebogang Gaogane, Mulenga M. Mukanu, Twalib Ngoma, Charles Mulindabigwi Ruhara, Milkah N Wanjohi, Karen Hofman

**Affiliations:** a Faculty of Health Sciences, SAMRC/Centre for Health Economics and Decision Science, PRICELESS, University of Witwatersrand School of Public Health, Johannesburg, South Africa; b Menzies Centre for Health Policy, School of Public Health, The University of Sydney, Sydney, Australia; c Economic Policy Research Centre (EPRC), Makerere University, Makerere, Uganda; d Faculty of Health Sciences, University of Namibia, Windhoek, Namibia; e African Population and Health Research Center, Health and Systems for Health Unit, Kenya; f Boitekanelo College, Department of Health Promotion & Education, University of Botswana, Gaborone, Botswana; g Health Policy and Management Unit, University of Zambia School of Public Health, Lusaka, Zambia; h Economic and Social Research Foundation, Muhimbili University of Health and Allied Sciences, Dar Es Salaam, Tanzania; i School of Economics, University of Rwanda, Butare, Rwanda

**Keywords:** Jennifer Stewart Williams, Non-communicable disease, Botswana, Kenya, Namibia, Rwanda, Tanzania, Uganda, Zambia

## Abstract

**Background**: Credible data and indicators are necessary for country-specific evidence to support the design, implementation, monitoring and evaluation of sugar-sweetened beverage (SSB) taxation.

**Objective**: A cross-country analysis was undertaken in seven Sub-Saharan African countries to describe the potential role of available data in strengthening SSB taxation. The objectives were to: document currently available data sources; report on public access; discuss strengths and limitations for use in monitoring SSB taxation; describe policy maker's data needs, and propose improvements in data collection.

**Methods**: The study used a mixed-methods approach involving a secondary data analysis of publicly available documents, and a qualitative exploration of the data needs of policy makers’ using primary data. Findings were synthesised and assessed for data strengths and weaknesses, including usability and availability. SSB taxation-related data availability was critically assessed with respect to adequacy in strengthening taxation policy on SSBs.

**Results**: Findings showed a paucity of SSB taxation-related data in all seven countries. National survey data are inadequate regarding the intake of SSBs and household expenditure on SSBs. Fiscal data from SSB tax revenue, value added tax from SSB sales, corporate income tax from SSB companies and SSB custom duty revenues, are lacking. Accurate information on the soft drink industry is not easily accessed.

**Conclusion**: Timely, easily understood, concise, and locally relevant evidence is needed in order to inform policy development on SSBs. The relevant data are drawn from multiple sectors. Cross- sector collaboration is therefore needed. Indicators for SSBs should be developed and included in current data collection tools to ensure monitoring and evaluation for SSB taxation.

## Background

Non-communicable diseases (NCDs) account for an estimated 40.5 million (71%) of the 56.9 million deaths worldwide, with the majority occurring in low- and middle-income countries (LMICs) [1]. Increasing prevention and control of NCDs is of particular importance in Sub-Saharan Africa (SSA) [2].

In 2017, high blood pressure, high blood sugar, high body-mass index (BMI) and dietary risks accounted for the greatest health losses from NCDs for both men and women in SSA [2]. Dietary risk factors contributed to 13 million disability adjusted life years (DALYs) and 489,000 deaths in SSA, of which 139,000 DALYs and 3,000 deaths were attributable to high consumption of sugar-sweetened beverages (SSBs) [3]. In 2017, the consumption of SSBs (49 grams per day) was almost twenty times higher than the optimal level of intake (3grams per day) across the whole of the SSA region [4]. With strong evidence about the adverse health effects of SSBs on population health [5], the taxation of SSBs has been identified as an entry point for creating healthier food environments [6]. The use of SSB taxation as a lever to improve population health and its efficacy as a tool to curb obesity, is often contested by the food and beverage industry. A growing body of literature on nutrition policies including SSB taxation, describes the critical role of data availability in increasing political and public support in policy implementation [7–9]. For example, in South Africa, strong local evidence and data demonstrating the potential impact of a 20% SSB tax on obesity was instrumental in supporting advocacy efforts and creating sustained political commitment for the adoption of a SSB tax [10].

An increasing number of LMICs are considering the implementation of SSB taxation or optimizing existing measures to align these with the global best-practice recommendation of 20% that would generate meaningful changes in consumption [11,12]. However a number of LMICs lack the confidence to act on SSBs in part due to the lack of reliable local and timely data on the effectiveness of such tax [13].

This article aims to describe the data availability and its potential role in strengthening SSB taxation in seven SSA countries. The objectives were to: document currently available data sources; report on public access; discuss strengths and limitations for use in monitoring SSB taxation; describe policy makers’ data needs and propose improvements in data collection.

## Methodology

### Study design and setting

This study has a mixed-methods design involving a secondary data analysis of publicly available documents, and a qualitative exploration of the data needs of policy makers using primary data. This is part of a larger research project, *Readiness to Adopt Inter-sectoral Non-Communicable Disease Prevention Policies in a Subset of Southern and East African Countries: A Landscape Analysis* [14]. The study reported in this paper was undertaken in Botswana, Kenya, Namibia, Rwanda, Tanzania, Uganda, and Zambia with the primary research question: What are the potential opportunities and challenges to strengthen SSB taxation-related policies? The research question is addressed by identifying relevant data and assessing data availability related to SSB taxation in these seven countries.

### Data collection

**Figure 1.**
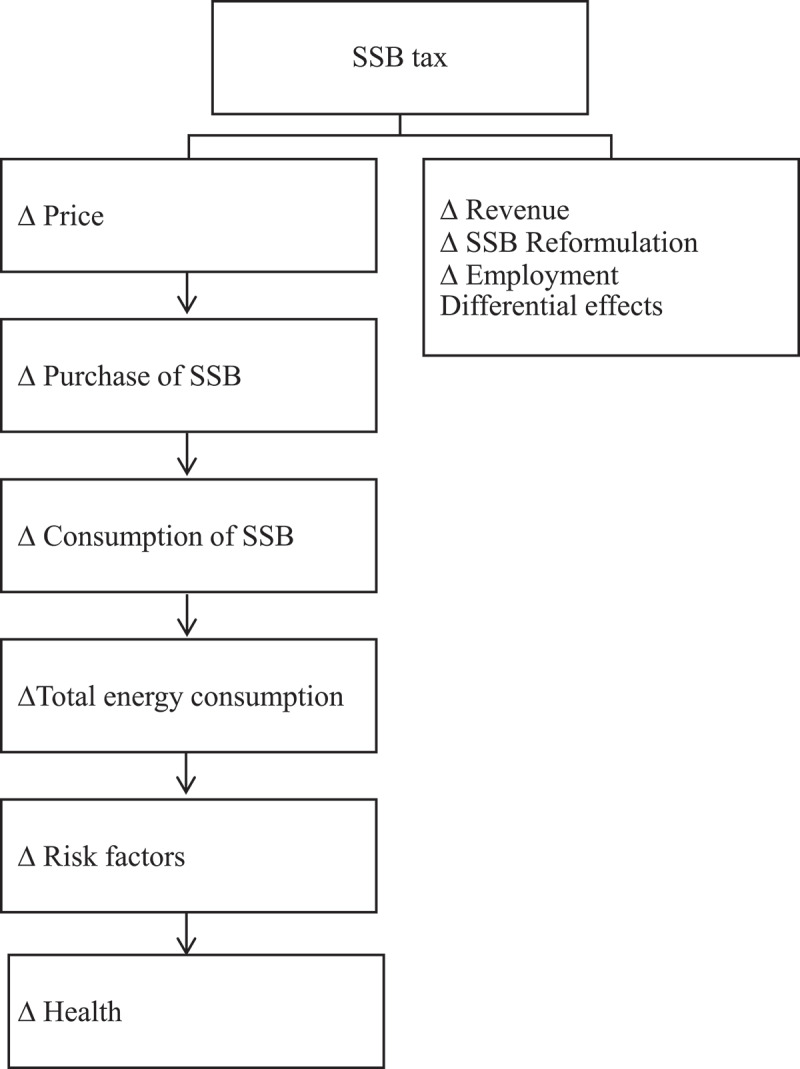
Summary of potential outcomes of SSB taxation. Adapted from Mytton, Eyles & Ogilvie, 2014 [15]

The research team collaboratively identified relevant indicators based on 1) the nature of SSB consumption as a health problem (i.e., indicators relevant to diet and health) and 2) the context of SSBs as a consumer good (i.e., related to supply chains). We consulted the broader literature on the theory of change of how SSB taxation can influence health [15,16] and how it can lead to additional outcomes that result directly from the tax (Figure 1).

For each potential effect of SSB taxation we brainstormed indicators that can be effective measurement tools. We organized them into the following groups: health, anthropometry, food, diet, economic, fiscal, employment specific indicators, and sociodemographic variables. Gender-sensitive data were also collected where available.

The secondary data analysis was undertaken between October 2018 and March 2019 to document currently available data sources, to report on their public availability, and to discuss data limitations and strengths. We searched for publicly available documents including surveys and routine data collection systems at national and certain sub-national levels with relevance for monitoring the impact of SSB taxation. Data sources included ministry reports (Health, Trade, Commerce, Agriculture, Education, Finance/budget statements), publications from government statistics offices, the World Bank, the World Health Organization (WHO) and other development partners such as the Food and Agriculture Organization of the United Nations (FAO). Researchers also looked for data at The Demographic and Health Surveys (DHS) Program website [https://dhsprogram.com/] and DataFirst [https://www.datafirst.uct.ac.za/] online data repository. In order to obtain industry relevant data, Fitch Solution [17] reports were purchased. These reports specifically deal with the food and drink markets in Botswana, Zambia, Namibia, Tanzania, Uganda and Kenya. No report of this type was available for Rwanda.

To understand the data needs of policy makers, primary data were collected through 39 key stakeholder interviews (KSI) conducted between October 2018 and April 2019 in four of the seven countries (10 interviews in Kenya, 10 in Zambia, 13 in Namibia and 6 in Botswana). Researchers (MM, HA, GA, LG) developed a stakeholder analysis matrix based on the recommendations of Varvasovsky and Brugha[18] . Stakeholders were selected based on 1) the desk-based policy content analysis, 2) the desk-based stakeholder analysis components of the parent research project. Stakeholders invited for a one-on-one interview included representatives from government agencies including health, commerce, development, agriculture, education; academia; soft drink manufacturers; and civil society actors (Table 1). Interviews were conducted by an experienced English language researcher. A semi-structured interview schedule was used as a guide. Among other issues, questions focused on the type and adequacy of data to support SSB taxation. All interviews were audio recorded and transcribed verbatim into English.

**Table 1.** Key stakeholder description

Stakeholder category	Country (number of stakeholders)
Government sector	Kenya (4) Namibia (9) Zambia (6) Botswana (3)
Civil society	Kenya (5) Namibia (1) Zambia (3) Botswana (2)
Industry	Zambia (1)
Academia	Kenya (1) Namibia (3) Botswana (2)

### Data analysis

#### Data availability landscape

Relevant data sources were assessed according to their strengths and weaknesses, including usability and availability, furthermore their characteristics including frequency of data collection, and geographical coverage. A standardised data collection template was developed on Microsoft Excel™ to facilitate data collection across the seven research sites. Thereafter, data were compared and synthesised during a workshop held in Johannesburg in South Africa in March 2019, involving researchers from each of the seven countries.

#### Analysis of key stakeholder interviews

Interview transcripts were read multiple times by two researchers in order to immerse in the content and flow of the discussion. Thereafter, transcripts were coded using a qualitative analysis tool – Nvivo version 12. The first round of the coding process was guided by objective three, to describe data needs of policy makers. The following codes were used in the analysis: data as a barrier, data to drive policy, data from other countries, data for monitoring and evaluation, data type, and local data. Thereafter, themes were identified through a deductive, data-driven coding approach, while the analysis took an inductive form simultaneously reflecting on the answers given to the research questions. Reporting of the findings adheres to COREQ guidelines DHS data primarily[19] .

#### Integrated analysis

Given the mixed-methods nature of our study, we integrated findings from across the data sets. We examined findings for convergence and contradiction to develop a coherent and cross-validated picture of data availability in the context of SSB taxation. By comparing these sources, we identified gaps in data availability and proposed improvements to data collection accordingly. This integration took place after the data analysis was completed.

### Ethical considerations

Ethical approval for conducting interviews was granted at each site. Ethical clearance was obtained by the Amref Health Africa – Ethical and Scientific Review Committee (Amref-ESRC- ESRC) in Kenya, the Research Ethics Committee at the University of Namibia, ERES Converge in Zambia, and Ministry of Health in Botswana.

## Results

### Data availability landscape

#### Existence of current surveys

Surveys are widely used data sources among researchers [20]. Survey data are imperative for robust analysis and to build knowledge base which is then made available to policy and programmatic decision-makers.

Table 2 summarises the availabile surveys in the study countries. The principal source of data on the NCD burden and associated risk factors is the WHO STEPwise approach to Surveillance (STEPS) [21]. STEPS, which was initiated in 2002, collects comparable information across countries on NCDs. These data are available in five of the seven countries. However, STEPS data are older than eight years in Tanzania (conducted in 2012). In Rwanda and Namibia where STEPS data are unavailable, the WHO NCD Country Profile consolidates data from several sources.

**Table 2.** Summary of the latest available country data for national assessment data indicators

Country	Demographic Health Survey (DHS)	Household survey	National nutrition survey (NNS)	STEPS	FAO Food Balance Sheet (FBS)
Botswana	1988	2015/16	-	2015	2013
Kenya	2014	2013	2018	2015	2013
Namibia	2013	2015/16	2014	-	2013
Rwanda	2014/15	2016/17	2018	-	2013
Tanzania	2016	2014/15	2014	2012	2013
Uganda	2016	2016/17	-	2014	2013
Zambia	2014	2015	2008	2017	2013

All seven countries collect national population-based data using DHS Surveys. However, the most recent Namibian and Zambian DHS reports are older than five years and Botswana conducted only one DHS in 1988. The latest Botswana Demographic Survey, conducted in 2017, does provide information on certain health indicators. DHS data primarily focus on health and is the most detailed source of local health data. The DHS surveys are also the principal source of data on nutrition-specific indicators including anthropometric measures and data on child feeding practices across all countries.

Routine surveillance is undertaken by the District Health Information Systems (DHISs) in all seven countries. The DHIS includes a number of NCD-related indicators such as diabetes, hypertension, mental health, dental health, cataract surgery and cervical cancer screening. The current DHIS data are not population based. They capture only those who seek care in the public sector.

Other surveys that provide data on food-related indicators (i.e., food consumption, food security) at a national-level are conducted on households. Table 2 outlines the availability of these and other data sources across countries. Across the countries, only Rwanda has district-level data in its Integrated Household Living Conditions Survey and the DHS. In the other six countries, the lowest-level of data available is at provincial-level. Among all surveillance tools, household surveys are conducted the most frequently, but their dependence on self-reported data makes these a limited source of health data. Data from surveys with a primary focus on nutrition were available in Tanzania, Kenya, Rwanda, Namibia and Zambia. National nutritional surveys were unavailable in Uganda and Botswana.

Country-level data relevant to food and beverages include food balance data collected by the FAO in all seven countries [22]. The latest data (2013) compiled by FAOSTAT provide measures on the total per-capita annual availability of particular foods for human consumption such as sugar, non-alcoholic beverages and fruit juices, taking into consideration agricultural production, imports, and exports in each country.

Regarding the soft drink industry data, annual reports and the media (both printed and digital) are provided free of charge, but there is limited information on products content, sales, and price. Alternative sources for industry information include independent data providers such as Euromonitor International and Fitch Solutions [17]. However, these independent data providers charge for their data and did not have industry information available in all of the seven countries studied here.

#### What is being monitored?

All countries have some nationally representative data on the prevalence of NCDs and survey data on nutrition status indicators from DHS. Core nutrition related anthropometric indicators monitored in all countries include BMI, stunting, wasting, raised blood pressure, overweight/obesity, raised blood glucose, raised cholesterol, and micronutrient deficiency. Importantly, the data are disaggregated by gender and age. A nutrition-relevant but under reported indicator is dental health, which was only monitored in Botswana and Zambia at the time of data collection (2019). Monitoring of other nutrition-related NCD indicators varies in scope across countries and even across surveys within a country depending on the primary objective of the survey. The nutritional focus of the surveys is dominated by questions around household food insecurity, dietary diversity, vitamin supplementation, breastfeeding, and infant and child feeding practices. Target populations of surveys are often restricted to the dietary patterns and health and nutrition outcomes of children 6–59 months of age and women of reproductive age 15–49 years old. This is the same for the Zambia Food Consumption and Micronutrient Survey and DHS data in Kenya, Rwanda, Tanzania, and Zambia.

Table 3 provides a detailed summary of the indicators relevant to SSB taxation that are captured by existing surveys in the respective countries. For an extended list of nutrition-related NCD indicators please see Appendix A.

**Table 3.** Summary of the availability of indicators in the studied countries that are relevant to support the formulation, implementation and evaluation of SSBs taxation

Domain		Botswana	Kenya	Namibia	Rwanda	Tanzania	Uganda	Zambia
Health	NCD prevalence (diabetes, CVD, hypertension, cancer)	x	x	x	x	x	x	x
	Dental health	x						x
	Child obesity		x	x	x	x	x	x
	Adult obesity	x	x	x	x	x	x	x
Anthropometry	BMI	x	x	x	x	x	x	x
Dietary behaviour	Consumption of sugar		x		x		x	x
	Consumption of sweets							x
	Added sugar to child’s food						x	
	Consumption of non-SSBs (soft drink, juice, soda)	x	x			x	x	x
	Consumption of SSBs	x	x					x
	Total dietary energy intake						x	x
Food	Food security status	x	x	x	x	x	x	x
	Household food sources	x	x	x	x		x	x
	Food prices		x	x	x	x	x	x
Economic	Sales volumes of sugar (quantity)	x		x	x	x	x	x
	Import/export quantity and value of non-alcoholic beverages	x	x	x	x	x	x	x
	Per Capita supply of sugar	x	x	x	x	x	x	x
	Sugar production		x	x	x	x	x	x
Socio-economic	Household healthcare expenditure	x	x	x	x	x	x	x
	Household food expenditure	x	x	x	x	x	x	x
	Household expenditure only on soft drinks (inc. water, juices, sodas)		x		x	x	x	
	Household expenditure only on SSBs				x			
Sof drink industry	Sales revenue	x	x	x	x	x	x	x
	Number of companies in industry sector	x		x	x	x	x	x
	Beverage industry forecasts	x	x	x		x	x	x
	Beverage price data				x	x	x	
	Package sizes currently available							
	Number of low/no-calorie beverages							
	Sugar content (gm of sugar/100ml)							
Fiscal	Food-related tax rates	x	x	x	x	x	x	x
	SSB customs duty rates			x	x	x	x	
	SSB custom duty revenue						x	
	General tax revenue (% of GDP)	x	x	x	x	x	x	x
	SSB tax revenue				x		x	
	VAT from SSB sales						x	
	VAT from substitute product sales							
	CIT from SSB companies							
	CIT from SSB substitute companies							
Employment	Employment by economic activity	x	x	x	x	x	x	x
	Employment in the SSB industry							

X indicates source available.

At an aggregate level, dietary risk factors that are monitored include fruit and vegetable intake, salt, sugar, meat, dairy products, and alcohol consumption. Data on non-alcoholic beverages (non-SSB specific) are available in Uganda, Tanzania, Zambia, and Kenya. The surveys often cluster a number of different items together ranging from soft drink, bottled/canned soda, juice, non-milk liquids and water. The DHS surveys in all countries assembled particular food types together. For example, non-milk liquids were grouped together when collecting data on child-feeding practices. Similarly, Tanzania’s national nutrition survey asks participants about purchasing and consuming any of the beverages listed, including SSBs and non-sugary beverages alike:

Any sodas or other sweet drinks, like Azam, Pepsi, Twist, local herbs, gripe water, clear tea with no milk, black coffee, togwa [traditional non-alcoholic beverage] (National Nutrition Survey, 2014, Tanzania).

Similar methods of categorisation were observed in other surveys in Tanzania, including the Household Budget Survey (2011/12) that provides household expenditure data on a sub-group of drinks that groups mineral waters, soft drinks, fruit and vegetable juices together; and the National Panel Survey (2014/15) that provides price data per kilogram for bottled/canned soft drinks, soda, juice, and water. The price of and expenditure on SSBs are also not disaggregated from non-sugary drinks in Kenya’s Integrated Household Budget Survey (2015/16). Here cost data are only available for non-alcoholic beverages in general. Rwanda’s Integrated Household Living Conditions Survey (2016/17) is the only tool that captures the share of household spending on carbonated soft drinks as a single item. Additional relevant items included in that survey included juices (local and imported), candy/gum, chocolate, powdered juices and drinking chocolate, sugar (local and imported).

Out of the seven SSA countries, only Kenya’s PMA2020 nutrition survey includes a recently added a specific question about the consumption of SSBs among women 10–49 years of age and children 6–59 months of age. An evaluation conducted on the survey found the question to be feasible and suggested that it should be included in nation-wide population-based surveys [23]. The PMA2020 as well as the surveys in Tanzania and Rwanda includes questions about diet (i.e., sweet and savoury/fried snacks consumption) that are not commonly measured in nationally representative household surveys.

### Data needs of key stakeholders

Results from the key stakeholder interviews are presented in alignment with four themes that emerged from the analysis: 1) context-specific NCD profiles, 2) sugar and SSB consumption data, 3) economic data, and 4) intervention effectiveness. Under each theme, results are supported by illustrative quotes.

#### Context-specific NCD profiles

Local evidence generation on priority nutrition-related NCDs and drivers was perceived necessary for policy formulation amongst key stakeholders.

If you don’t have evidence, the people who need to make the change are not going to make the change that you need. (KSI, Kenya, policy maker)

There was a strong reliance on external surveys conducted by outside agencies rather than on internal data, which raised concerns among stakeholders regarding the applicability of the data to local context. A policy maker in Botswana emphasised the need to identify local drivers of NCDs to inform how best to adapt generalised, international best practice to local contexts and target true drivers of NCDs.

We need enough data on the real causes of NCDs in Botswana. Internationally or globally one of the main contributing factors of obesity - also linked to NCDs - is sugar intake. However, the same cannot be assumed for Botswana without adequate evidence. (KSI, Botswana, dietician)

Similarly, in Kenya a concern was expressed regarding how the calorie dense nature of local diets may not be captured by the standardized global NCD surveys.

We have a big problem of content. Our food now is very calorie dense. Then we have another challenge when we talk about NCDs, [the] combination of our diet, very few vegetables and fruits and so we have a lot of starch and protein. (KSI, Kenya, policy maker)

Interviewees unanimously recommended introducing monitoring and data collection aimed at better understanding local diets and context-specific foods and beverages. For example, in Zambia, six of the ten interviewees believed that Nshima, a common processed mealie-based breakfast food, might contribute to NCDs. In Namibia, a stakeholder expressed concern over the high sugar content of home brewed beverages. A participant from the government sector in Botswana spoke more generally about indigenous and locally produced foodstuff and the need to better monitor and evaluate these health impacts.

Furthermore, stakeholders believed that data disaggregated by district, as opposed to current national or provincial-levels, would allow the identification of hot spots for particular public health attention. One interviewee from Namibia described the different dietary habits across the counnationaltry, variation that might not be captured in great depth by existing surveys.

If we start from the North, we have mostly people who are emphasizing on meat derived products. In the northeast, there’s more focus on wild edible food like fruits and vegetables together with the game meat. If we come to the more advanced and industrial society in the central regions in Namibia, we find most of the people focus on the industrialised or value-added foods maybe purchased from supermarkets. (KSI, Namibia, Researcher)

#### Sugar and SSB consumption data

When asked about the type of evidence needed for the successful implementation of a tax on SSBs, interviewees hoped for high-quality data on the amount of sugar consumed per capita and the amount of sugar in particular foods.

We need evidence on diet habits of the population and then based on the evidence we will also establish that indeed there is a higher consumption of sweetened foods or higher consumption of sugary foods or higher consumption of fatty foods. (KSI, Namibia, civil society)

Baseline in terms of nutrition we have, but in terms of how much sugar, because we say that coca cola has this sugar but because we have never had evidence. […] The ready-made products even from supermarket even the cereals we don’t have evidence of sugars and salts that go in. (KSI, Kenya, policy maker)

Demographic information of consumers was also highlighted as being important data for policy makers. When asked about national-level data deficits with regards to nutrition, interviewees indicated that men and the adult population were less represented in the survey data.

Research is really lacking, most of the research is on under-fives, pregnant women but very few on adults. (KSI, Botswana, dietician)

Beyond age and gender, some interviewees identified the value for policy making of having data on socio-economic status and consumers` motivation for their food and drink choices.

Who are the consumers of the same drinks we are talking about? The people who are in danger, the elite: the people who went to school, the middle class? (KSI, Zambia, policy maker)

What type of food do they have even as related to their income etc. and the consumption of sugar and the type of food they eat and the reason why they are eating such foods, is it lifestyle, is it health, is it culture. (KSI, Namibia, policy maker)

#### Economic data

Stakeholders emphasised the need for SSB-related fiscal evidence to compare the cost and the potential benefits of the proposed SSB tax. Equally, economic evidence was perceived to be key for the evaluation of any current or potential tax and its augmentation.

The research would be to bring out the economic cost and say how much do we lose, how much do we gain. How much of tax are we gaining vis-à-vis how much are we using from these diseases. And also to look at the cost of putting the tax. […] How much revenue do you gain as a government, how much reduction of consumption, how much does it affect the work industry. (KSI, Kenya, policy maker)

Some also noted that information on the soft drink industry would be useful for policy making, and balancing health and economic priorities. For example, one participant raised the question *‘how many factories do we have in the country’ (KSI, Namibia, policy maker)*. Furthermore, obtaining accurate information on the price of foods and product content, emerged as an important discussion point.

#### Intervention effectiveness

One interviewee furthered the discussion by calling for evidence with nation-wide geographical coverage and longitudinal time. This was perceived to be important for the development and implementation of a SSB tax.

You need proven evidence. When the UK introduced [a policy on SSBs], they done a study for 25 years. They looked at several parameters after that they said no, not anymore, then it [a policy on SSBs] was introduced and approved by the parliament. (KSI, Namibia, researcher)

Similar country examples of successful implementation of SSB taxation were deemed invaluable by interviewees claiming that ‘*it would also be interesting for the policy makers to understand what is happening in other countries so that they could extrapolate and see how it is going to benefit our own local population’ (KSI, Kenya, policy maker)*. However, there was uncertainty about the transferability of evidence from other countries. Therefore, interviewees called for local evidence generation.

To introduce tax based mechanism to reduce the consumption, I think clinically based proven studies with big number of case studies that is to be considered, maybe from different regions in Namibia to be introduced from the Ministry of Health together with the Ministry of industry and trade or then to be taken to the cabinet in Namibia to highlight […] that we are getting lots of patients and this is killing our people (KSI, Namibia, researcher)

Similarly, in Botswana, a dietician acknowledged that a SSB tax ‘*is an international recommendation but for Botswana I don’t think it’s adequate’ and* called for local evidence generation in the form of a pilot study to provide an evidence base for a tax proposal on SSBs.

### Integrated analysis of gaps and opportunities

By using both secondary data analysis and qualitative methods this study sought to provide a cross-validated picture of data availability for the successful monitoring and evaluation of SSB taxation in seven SSA countries.

Our findings highlight a data deficit in the following areas: SSB consumption and purchasing behaviour, economics, and industry matters. Sociodemographic variables cut across all of these areas. We found minimal sugar consumption data at the individual-level. Neither the national surveys, nor the DHS or FAO explicitly assess the consumption of SSBs. There is also a dearth of household-level expenditure data on SSBs. Yet these are necessary indicators for making estimates of consumption estimates and formulating policy. We found very little SSB fiscal data or publicly available information on industry trade and sales volumes. Although annual industry reports do exist, they are not easily accessible for the general public.

Findings from the secondary analysis and stakeholder interviews highlighted significant data gaps in relation to age and gender. Most of the nutrition data collected to date has been for children aged under-two and for women of reproductive age. Groups that were less represented included children aged 5–9 and 10–14 years and men in general. Furthermore, differences between urban and rural residents, richer and poorer groups were not documented.

## Discussion

The current data landscape in these seven countries provides only a snapshot of nutrition-related NCD risk factors and offers little data to support the design, implementation, monitoring and evaluation of SSB taxation in SSA.

The deficits of current nutrition-related indicators in large-scale household and facility surveys have also emerged as a recent concern in global health circles. In 2018, an international technical consultation on monitoring nutrition-related NCDs, highlighted the urgent need for new indicators on unhealthy diets, including SSB consumption [24]. More recently, a review of data availability on children’s diets highlighted large data gaps in LMICs regarding unhealthy dietary practices across all age groups [25]. The review’s recommendations resonated with that of the 2018 technical consultation report [24] and also our study findings – to strengthen standardized, nationally representative individual-level dietary and food consumption data, including SSB intake. In the absence of such evidence, countries might underestimate the true magnitude of SSB consumption. Evidence that reflects sociodemographic differences is also imperative for policy implementation and evaluation. Sociodemographic data can help inform researchers about the economic regressivity of taxation policies and can assist in evaluating the effects of pricing on consumer behaviour. Coherent economic arguments must also be used as a part of a comprehensive approach to discuss SSB taxes within policy circles. Key stakeholders unanimously underscored the importance of such evidence that policy makers need to take into account.

While no consensus has been reached to date around how best to capture data for new indicators [24], a useful point of departure would be to complement existing data sources, including population-based surveys with SSB taxation-related questions. This would lead to improvement in tracking SSB intake and the effectiveness of SSB taxation. Surveys like the DHS could include specific questions on SSB and added sugar consumption, while GHS and National Income Dynamics Studies could include questions around SSB spending. However, household surveys and their modification are costly and would require investments. National nutrition surveys in Burkina Faso and Tanzania can cost between 25–45 USD per household, 15–23,000 USD per stratum per year respectively [24]. National Departments of Health should allocate funds to improve data surveillance processes and convene relevant stakeholders, including data producing agencies and local research groups, with the aim of synchronising data sources. This is necessary, as data are often held across various sectors e.g. economic, labour and health, and by government and non-governmental organizations as well as by various parts of food and beverage industries. A growing body of literature on multi-sectoral public health policy suggest that successful interventions require a collaborative approach to gather evidence [26–28]. In order to achieve this, all relevant sectors should coordinate their efforts. Transparent and collaborative efforts would enable the most efficient use of various data sources.

## Strength and limitations

This analysis fills a gap, for the first time, in the data available regarding SSB consumption and taxation in Sub-Saharan Africa. The analysis has a few limitations, however. First, key stakeholder interviews were only conducted in four of the seven countries due to budget constraints. Nevertheless, stakeholder interviews provided indicative evidence on the type of evidence they require for strengthening SSB taxation. Second, the desk review relied heavily on publicly available online material, thus recent unpublished data might have been excluded. To ensure that all key data sources have been included in our analysis, stakeholder interviews served as means of verification. This study represents a first step towards global benchmarking of SSB taxation-related data against which countries can compare their progress in improving data availability.

## Conclusion

Diets in SSA are transitioning as countries advance from low-income to middle-income status. It is timely to adopt preventive measures to curtail the growing NCD epidemic. One important area of focus is the SSA food and beverage space. Currently SSA is being promoted as a ‘growth market’ by the food and beverage industry. Fiscal policies, such as SSB taxation could have substantial health benefits in the long term. Yet they are one policy lever in a series of steps that is needed to address population health and obesity prevention. The potential health and economic benefits of such tax as well as the monitoring and evaluation of its implementation will require appropriate data and indicators within and beyond the health sector. The establishment of robust accurate baseline data to inform evidence will enable governments to accelerate political and public support for SSB taxation and related policies.

## Paper context

In the face of the high non-communicable disease burden across Sub-Saharan Africa, sugar-sweetened beverage taxation could have substantial health and economic benefits. Its success requires credible data to inform a strong evidence base. This study enhances the understanding of data availability and missing information required for strengthening sugar-sweetened beverage taxation in Botswana, Kenya, Namibia, Rwanda, Tanzania, Uganda, and Zambia. This study contributes to knowledge of appropriate design, successful implementation and evaluation of sugar-sweetened beverage taxation across Sub-Saharan Africa.

## Appendix A. Summary of available data on nutrition-related NCDs and risk factors in seven SSA countries

**Table:**

Domain		Botswana	Kenya	Namibia	Rwanda	Tanzania	Uganda	Zambia
Health	NCD prevalence	BDS 2017, STEPS 2015	STEPS 2015	WHO 2016	WHO 2016	STEPS 2012	STEPS 2014	LCMS 2015, STEPS 2017
	Diabetes	BDS 2017, STEPS 2015	STEPS 2015	DHS 2013, WHO 2016	WHO 2016	STEPS 2012	STEPS 2014, UNHS 16/17	LCMS 2015, STEPS 2017
	Cardiovascular disease	BDS 2017, STEPS 2015	STEPS 2015	WHO 2016	WHO 2016	STEPS 2012	STEPS 2014	LCMS 2015, STEPS 2017
	Hypertension	BDS 2017, STEPS 2015	STEPS 2015	DHS 2013	WHO 2016	STEPS 2012	STEPS 2014	LCMS 2015, STEPS 2017
	Micronutrient deficiency	BDS 2017	DHS 2014 KNMS 2011	DHS 2013	DHS 2014/15	DHS 2015/16	DHS 2016	LCMS 2015, FCMS 2014
	Cancer	BDS 2017, STEPS 2015	STEPS 2015	WHO 2016	WHO 2016	STEPS 2012	STEPS 2014	LCMS 2015, STEPS 2017
	Dental health	BDS 2017						LCMS 2015
	Mental health	BDS 2017		DHS 2013				STEPS 2017
	Child obesity		DHS 2014	DHS 2013	DHS 2014/15	DHS 2015/16, NNS 2014	DHS 2016, STEPS 2014	DHS 2013/14, STEPS 2017
	Adult obesity	BDS 2017, STEPS 2015	DHS 2014, STEPS 2015	DHS 2013, WHO 2016	DHS 2014/15, WHO 2016	DHS 2015/16, NNS 2014, STEPS 2012	DHS 2016, STEPS 2014	DHS 2013/14, STEPS 2017
Anthropometry	BMI	BDS 2017	DHS 2014	DHS 2013	CFSVA 2018, DHS 2014/15	NNS 2014,DHS 2015/16	DHS 2016	FCMS 2014
	Stunting	BFHS 2007/08	DHS 2014	DHS 2013	CFSVA 2018, DHS 2014/15	NNS 2014, NPS 2014/15, DHS 2015/16	DHS 2016, NPS 2015/16	LCMS 2015
	Wasting	BFHS 2007/08	DHS 2014	DHS 2013	CFSVA 2018, DHS 2014/15	NNS 2014, NPS 2014/15, DHS 2015/16	DHS 2016	LCMS 2015
	Total cholesterol	STEP 2015	STEPS 2015			STEPS 2012	STEPS 2014	STEPS 2017
Dietary behaviour	Fruits and vegetables (all ages)	BDS 2017, STEP 2015	STEPS 2015	DHS 2013	CFSVA 2018	NNS 2014, STEPS 2012	UNHS 16/17, NPS 2015/16	FCMS 2014
	Salt (all ages)	STEPS 2015	STEPS 2015	WHO 2016	WHO 2016	NNS 2014, STEPS 2012	UNHS 16/17, NPS 2015/16	FCMS 2014
	Fat (all ages)		STEPS 2015		CFSVA 2018	STEPS 2012	UNHS 16/17, NPS 2015/16	FCMS 2014
	Sugar (all ages)		STEPS 2015		CFSVA 2018		UNHS 16/17, NPS 2015/16	FCMS 2014
	Alcohol (all ages)	STEP 2015	STEPS 2015	DHS 2013, WHO 2016	WHO 2016	DHS 2015/16, STEPS 2012	UNHS 16/17, NPS 2015/16	STEPS 2017, FCMS 2014
	Sweets (all ages)							FCMS 2014
	Vitamin supplementation		DHS 2014	DHS 2013	CFSVA 2018, DHS 2014/15	NNS 2014, DHS 2015/16	DHS 2016	FCMS 2014
	Breastfeeding	BDS 2017	DHS 2014	DHS 2013, NFNSM 2016	CFSVA 2018, DHS 2014/15	NNS 2014, DHS 2015/16	DHS 2016	FCMS 2014
	Infant and child feeding practices	BDS 2017	DHS 2014	DHS 2013	CFSVA 2018	NNS 2014, DHS 2015/16	NPS 2015/16	FCMS 2014
	Added sugar to child’s food						NPS 2015/16	
	Consumption of non-SSBs (soft drink, juice, soda) (all ages)	STEPS 2015	STEPS 2015			NNS 2014	UNHS 16/17, NPS 2015/16	FCMS 2014
	Consumption of SSBs (all ages)	STEPS 2015	STEPS 2015					STEPS 2017
	Total dietary energy intake						UNHS 16/17	FCMS 2014
	Consumption of added sugar (all ages)		STEPS 2015					
Food	Food security status	MTHS 2015/16	DHS 2014	NFNSM 2016	CFSVA 2018	NPS 2014/15	UNHS 16/17	FCMS 2014
	Dietary diversity		DHS 2014	NHIES 2015/16	CFSVA 2018	NPS 2014/15	UNHS 16/17	FCMS 2014
	Meal frequency		DHS 2014	NHIES 2015/16	CFSVA 2018	NPS 2014/15	UNHS 16/17	FCMS 2014
	Meal adequacy		DHS 2014	NHIES 2015/16	CFSVA 2018	NPS 2014/15	UNHS 16/17	FCMS 2014
	Household food sources		HBS 2015/16	NFNSM 2016	CFSVA 2018, IHLCS 2016/17		UNHS 16/17	LCMS 2015
	food prices		HBS 2015/16	NFNSM 2016	CFSVA 2018	NPS 2014/15	Consumer Price Index	LCMS 2015
Economic	Sales volumes of sugar (quantity)				IHLCS 2016/17		FBS	
	Import/export quantity and value of non-alcoholic beverages	FBS	FBS	FBS	FBS	FBS	FBS	FBS
	trade volumes (exc. Sugar, beverages)							
	Per Capita supply of sugar	FBS	FBS	FBS	FBS	FBS	FBS	FBS
	Domestic supply of sugar (imp.exp. Prod.)	FBS	FBS	FBS	FBS	FBS	FBS	FBS
	Food production	FBS	FBS	FBS	FBS	FBS	FBS	FBS
	Sugar production		FBS	NFNSM 2016	CFSVA 2018, IHLCS 2016/17, FBS	FBS	FBS	FBS
Socio-economic	Household healthcare expenditure	MTHS 2015/16	HBS 2015/16	NHIES 2015/16, DHS 2013	CFSVA 2018	NPS 2014/15, DHS 2015/16, HBS 2011/12	UNHS 16/17, NPS 2015/16	LCMS 2015
	Household food expenditure	MTHS 2015/16	HBS 2015/16	NHIES 2015/16, DHS 2013	CFSVA 2018	HBS 2011/12	UNHS 16/17, NPS 2015/16	LCMS 2015
	Household expenditure only on soft drinks (inc. water, juices, sodas)		HBS 2015/16		IHLCS 2016/17	HBS 2011/12	UNHS 16/17, NPS 2015/16	
	Household expenditure only on SSBs				IHLCS 2016/17			
Soft drink industry	Sales revenue	Fitch Solutions’ Food & Drink Report, Sechaba Brewery Holdings Limited Annual Report (2018)	FitchSolutions’ Food & Drink Report,	FitchSolutions’ Food & Drink Report	BralirwaLimited Annual Report 2018	FitchSolutions’ Food & Drink Report,	FitchSolutions’ Food & Drink Report	FitchSolutions’ Food & Drink Report, Zambia Breweries annual report, Varun beverages Zambia ltd
	Number of companies in industry sector	Sechaba Brewery Holdings Limited Annual Report (2018)		Fitch Solutions’ Namibia Food & Drink Report	Bralirwa Limited Annual Report 2018	Fitch Solutions’ Food & Drink Report	Fitch Solutions’ Food & Drink Report	Fitch Solutions’ Food & Drink Report, Zambia Enterprise map
	Beverage industry forecasts	Sechaba Brewery Holdings Limited Annual Report (2018) Fitch Solutions’ Food & Drink Report	Fitch Solutions’ Food & Drink Report	Fitch Solutions’ Food & Drink Report		Fitch Solutions’ Food & Drink Report	Fitch Solutions’ Food & Drink Report	Fitch Solutions’ Food & Drink Report, Zambia Food and Beverages, Zambia enterprise map
	Beverage price data				Bralirwa Limited Annual Report 2018		UNHS 16/17, NPS 2015/16	
	Package sizes currently available							
	Number of low/no-calorie beverages							
	sugar content (gm of sugar/100 ml)							
Fiscal	Food-related tax rates			PWC	PWC	PWC	PWC, URA	
	SSB customs duty rates				Deloitte	PWC	PWC	
	SSB custom duty revenue						URA	
	General tax revenue (% of GDP)	World Bank	World Bank	World Bank	World Bank	World Bank	World Bank	World Bank
	SSB tax revenue						URA	
	VAT from SSB sales						URA	
	VAT from substitute product sales							
	CIT from SSB companies							
	CIT from SSB substitute companies							
Employment	Employment by economic activity	MTHS 2015/16		DHS 2013		DHS 2015/16	DHS 2016 UNHS 16/17, NPS 2015/16	LCMS 2015, FCMS 2014
	Employment in the SSB industry							
Health promotion	Public expenditure							
